# P092 Pleural and pericardial effusions as the first manifestation of giant cell arteritis: a diagnostic challenge

**DOI:** 10.1093/rap/rkag113.122

**Published:** 2026-11-03

**Authors:** Shelter Bakah, Salema Khalid

**Affiliations:** Royal Bournemouth Hospital, Bournemouth, United Kingdom; Basingstoke and North Hampshire Hospital, Basingstoke, United Kingdom

## Abstract

**Introduction:**

Giant cell arteritis (GCA) is a chronic granulomatous vasculitis affecting the aorta and its major branches. It is the commonest primary vasculitis in adults over the age of 50 years. Typical manifestations include headache, constitutional symptoms, jaw claudication, visual disturbance and temporal artery abnormalities on examination. However, the clinical spectrum is broad, and less common manifestations involving the pericardium, respiratory and nervous systems have been described. We present a case in which pleural and pericardial effusions were the initial manifestations of GCA in the absence of cranial symptoms. Such presentations can pose a significant diagnostic challenge and delay appropriate treatment.

**Case description:**

A 60-year-old woman with no known comorbidities presented with difficulty raising her right arm, initially managed as subacromial bursitis with an ultrasound-guided corticosteroid injection. Over the following months, she developed recurrent chest, neck and interscapular pain with flu-like symptoms, resulting in multiple hospital attendances. Initial diagnoses included gastro-oesophageal reflux disease and pneumonia.

Four months later, she was admitted with chest pain and persistently raised inflammatory markers. CT pulmonary angiography demonstrated bilateral pleural effusions and a pericardial effusion without pulmonary embolism. She was treated for pleuropericarditis with antibiotics, NSAIDs, colchicine and prednisolone, with initial clinical improvement. However, over the following months she experienced recurrent symptoms, including interscapular pain, with rising inflammatory markers during repeated attempts to taper corticosteroids, resulting in multiple hospital attendances. Peak C-reactive protein (CRP) was 348 and erythrocyte sedimentation rate (ESR) was 91 alongside normocytic anaemia with haemoglobin of 107.

Extensive investigations for infection, malignancy and autoimmune disease, including blood cultures, tuberculosis screening, viral serology, connective tissue disease and ANCA testing, pleural fluid analysis and repeated CT imaging, were unrevealing. FDG-PET scan demonstrated subtle uptake within the thoracic aortic wall suspicious for vasculitis, together with metabolically active pleural and pericardial inflammatory effusions. Reduced scan sensitivity due to prior corticosteroid therapy was noted. Methotrexate was commenced, with leflunomide added because of recurrent disease flares. Methotrexate was later discontinued because of declining renal function. During her disease course, she developed an extensive proximal lower limb deep vein thrombosis despite a negative antiphospholipid antibody screen.

More than a year after diagnosis, she relapsed shortly after discontinuing the final 1 mg of prednisolone, with recurrent symptoms and rising inflammatory markers, highlighting the marked steroid sensitivity of her disease. Repeat FDG-PET demonstrated increased inflammatory uptake involving the thoracic aorta and proximal subclavian arteries, confirming active large-vessel vasculitis. Following multidisciplinary discussion, tocilizumab was initiated.

**Discussion:**

GCA is a heterogeneous disease comprising distinct cranial and large vessel phenotypes. Patients with large vessel involvement frequently present without the classical cranial manifestations of disease, contributing to diagnostic delay. Pleural and pericardial involvement are recognised but uncommon manifestations of GCA, and simultaneous pleural and pericardial effusions as the presenting feature is rare. When they occur in the absence of headache, jaw claudication or visual disturbance, infection, malignancy and connective tissue disease are more commonly considered, delaying recognition of GCA.

Our patient underwent extensive investigations and was reviewed by multiple specialties before the diagnosis of giant cell arteritis with large vessel involvement was established.

The clinical course provided important diagnostic clues. Recurrent pleural and pericardial effusions, persistently elevated inflammatory markers and repeated relapse during steroid tapering suggested ongoing systemic inflammation despite an initially inconclusive diagnostic work-up.

The case was discussed at our regional rheumatology meeting, where the diagnosis remained uncertain because of the atypical presentation and subtle PET findings. Continued clinical suspicion prompted a repeat FDG-PET scan during relapse, which demonstrated progression of inflammatory uptake involving the thoracic aorta and proximal subclavian arteries, confirming active large vessel vasculitis.

Our patient’s absence of cranial symptoms and recurrent relapses during steroid tapering are in keeping with previous studies demonstrating that large vessel involvement in GCA represents a distinct clinical phenotype associated with fewer cranial manifestations and a higher risk of relapse.

This case emphasises the importance of recognizing atypical presentations in GCA, particularly in patients with unexplained pleural and pericardial effusions in the absence of cranial symptoms. Early clinical suspicion and confirmation of the diagnosis may reduce repeated hospital admissions, prolonged corticosteroid exposure and diagnostic uncertainty, while enabling timely initiation of biologic therapy.

**Key learning points:**

1. Think about GCA in patients with persistently raised CRP/ESR and unexplained pleural or pericardial effusions that are steroid responsive, even in the absence of typical manifestations of GCA such as headache, jaw claudication or visual symptoms. This is something not only rheumatologists but also general physicians should keep in mind to avoid delays in diagnosis.

2. Where possible, FDG-PET should be performed before corticosteroid therapy is commenced, as steroid treatment may reduce scan sensitivity and increase diagnostic uncertainty, particularly in atypical presentations.

3. Persistent clinical suspicion despite inconclusive initial investigations should prompt reconsideration of the diagnosis. Repeat FDG-PET imaging during relapse may demonstrate active vasculitis and support timely escalation of therapy.

4. Patients with predominant large vessel involvement may have higher relapse rates and marked corticosteroid dependence despite conventional immunosuppressive therapy. These features should prompt reassessment of disease activity and consideration of treatment escalation.

